# On the Energy Contributions Driving Pyridine Adsorption on Silver and Gold Nanoparticles

**DOI:** 10.3390/nano15221720

**Published:** 2025-11-13

**Authors:** Tommaso Giovannini

**Affiliations:** Department of Physics, University of Rome Tor Vergata, Via della Ricerca Scientifica 1, I-00133 Rome, Italy; tommaso.giovannini@uniroma2.it

**Keywords:** energy decomposition analysis (EDA), nanoparticles, molecule–metal interactions, SERS, plasmonics

## Abstract

Understanding molecule–nanoparticle interactions is essential for theoretically describing the adsorption process. Here, we employ Kohn–Sham Fragment Energy Decomposition Analysis (KS–FEDA) to dissect the physical components driving pyridine adsorption on silver and gold nanoparticles. KS–FEDA is rooted in Density Functional Theory (DFT) and partitions the total energy into fragment-localized contributions, providing a rigorous decomposition into electrostatics, exchange–repulsion, polarization, dispersion, and exchange–repulsion terms. This framework offers a chemically intuitive interpretation of molecule–metal bonding at the DFT level, and for analyzing and parameterizing interactions at metal–molecule interfaces. The results highlight the relevant role of electrostatics and induction at localized sites and of dispersion over extended facets.

## 1. Introduction

The adsorption of molecular systems on noble-metal nanoparticles (NPs) is a topic of central importance in surface science, catalysis, and nanomaterial design [1,2,3,4,5,6]. Among the various metals exploited in these contexts, silver (Ag) and gold (Au) represent particularly compelling substrates because of their unique optical and electronic properties, including their plasmonic behavior [7,8,9,10,11]. Their ability to sustain localized surface plasmon resonances (LSPRs) enables a broad range of technological applications, from sensing [12,13,14,15] and photothermal conversion to photocatalysis [16,17,18,19,20,21]. The plasmonic response of these metals arises from the interplay between localized and delocalized charge carriers and interband transitions [22,23], and is highly sensitive to shape, size, and atomic-scale defects [24,25,26,27,28,29,30,31,32]. These structural features directly modulate the local electromagnetic enhancement that governs surface-enhanced spectroscopies [33,34,35,36,37,38], in particular, surface-enhanced Raman scattering (SERS) [39,40,41,42,43,44,45,46]. A quantitative understanding of how molecular adsorption modifies the energetics and electronic structure at the metal interface is essential to connect atomic-scale interactions with the measured optical response.

Theoretical approaches can shed light on the nature of molecule–metal interactions by disentangling the different physical components that contribute to binding. Various energy decomposition analysis (EDA) frameworks [47,48,49,50,51,52,53,54,55,56,57,58,59,60,61,62,63,64], e.g., Kitaura Morokuma-EDA (KM-EDA) [47], symmetry-adapted perturbation theory (SAPT) [52,53,54], and LMO-EDA [55,56,57,58], have been developed and successfully applied to characterize intermolecular interactions in both molecular and condensed-phase systems [58,65]. These approaches generally provide a chemically transparent breakdown of the total interaction energy into intuitive physical terms such as electrostatics, exchange (Pauli) repulsion, polarization, and dispersion. However, their direct application to molecule–metal interfaces remains limited [66,67,68].

In this work, we employ the recently developed Kohn–Sham Fragment Energy Decomposition Analysis (KS–FEDA) method [69]. KS–FEDA is rooted in density functional theory (DFT) and partitions the self-consistent Kohn–Sham energy into fragment-localized molecular orbital (KS-FLMO) contributions [69,70,71,72,73], enabling a rigorous separation of electrostatic, exchange–repulsion, correlation, and electronic-preparation terms. Dispersion interactions [74,75,76,77,78,79] can be incorporated through explicit nonlocal [80] or parametric (e.g., Grimme’s methods [81,82,83,84,85,86]) corrections. The method is robust when using diffuse and polarized basis sets [69], and its formulation allows direct comparison with symmetry-adapted perturbation theory (SAPT) results [52,53]. Remarkably, for molecular systems, KS–FEDA achieves an excellent agreement with high-level SAPT reference data (such as the golden standard SAPT2+(3)*δ*MP2) at a much lower computational cost [69].

KS–FEDA is applied here to the adsorption of pyridine on noble-metal nanoparticles. Pyridine is selected as it was the first molecule for which SERS was experimentally observed [41,42,43] and represents a prototypical adsorbate for coinage-metal surfaces [40,87,88,89,90]. Its simple structure, well-defined binding geometry through the nitrogen atom, and relevance to molecular spectroscopy make it an ideal benchmark for testing theoretical descriptions of molecule–metal bonding. Furthermore, by exploiting model systems for the NPs, such as minimal Ag_20_ and Au_20_ clusters [40,87,88,89,90], the essential physics of extended surfaces and adatom-like interactions can be captured, while remaining computationally accessible, thereby providing a robust framework for evaluating the KS–FEDA decomposition across different metals and adsorption motifs.

The paper is organized as follows: In the next section, we briefly recall the theoretical basis of KS-FEDA and KS-FLMOs. Then, we present the numerical results for Py-Ag and Py-Au systems by discussing the basis set convergence and a direct comparison with SAPT results and literature data. Finally, a summary and the conclusions end the manuscript.

## 2. Methods

The interaction energy between pyridine and metal nanostructures is decomposed by using the recently developed Kohn–Sham Fragment Energy Decomposition Analysis (KS-FEDA) [69]. KS-FEDA is based on the localization of KS Molecular Orbitals (MOs) on a specific pre-defined spatial region by means of KS fragment localized MOs (KS-FLMOs) [69,70,71]. Both methods, KS-FLMOs and KS-FEDA, are recalled in this section.

### 2.1. Kohn–Sham Fragment Localized Molecular Orbitals

Let us start our discussion with the DFT energy for a range-separated hybrid functional:

(1a) $$\begin{array}{cc} E[D]= & \mathrm{Tr}\;hD+\frac{1}{2}\mathrm{Tr}\;DJ\left(D\right)-\frac{1}{2}c_{x}\mathrm{Tr}\;DK\left(D\right)\\ & +\left(1-c_{x}\right)E_{x}\left[D\right]+E_{c}\left[D\right] \end{array}$$

(1b) $$=\mathrm{Tr}\;\;hD+\frac{1}{2}\mathrm{Tr}\;\;DJ\left(D\right)-\frac{1}{2}\mathrm{Tr}\;\;DK\left(D\right)\;\;+\left(1-c_{x}\right)\left(E_{x}\left[D\right]-E^{HF,x}\left[D\right]\right)+E_{c}\left[D\right]$$

where h is the one-electron matrix, while J and K are the Coulomb and exchange matrices, respectively. The matrix D represents the one-particle density matrix in the atomic orbital (AO) basis $\chi_{\mu }$, from which the electron density ρ(r) is obtained. The DFT energy functional is characterized by the exchange and correlation components, $E_{x}$ and $E_{c}$, while $E^{\mathrm{HF},x}\left[D\right]$ corresponds to the exact Hartree–Fock exchange energy. Pure and hybrid DFT functionals are defined by $c_{x}=0$ and $c_{x}\neq 0$, respectively. In Equation (1b), we exploit the Generalized Kohn–Sham (GKS) formalism [91,92], which allows us to include nonlocal exchange–correlation effects based on global hybrids, or range-separated or double-hybrid functionals [92]. The exchange and correlation energy contributions introduced in Equation (1) can be explicitly written as

(2a) $$\begin{array}{cc} \left(1-c_{x}\right)E_{x}\left[D\right] & =\left(1-c_{x}\right)\int \rho \left(r\right)\varepsilon_{x}\left(\rho \left(r\right)\right)\;dr-\frac{\beta }{2}\mathrm{Tr}\;DK^{\mathrm{LR}}\left(D\right) \end{array}$$

(2b) $$\begin{array}{cc} E_{c}\left[D\right] & =\int \rho \left(r\right)\varepsilon_{c}\left(\rho \left(r\right)\right)\;dr \end{array}$$

where $K^{\mathrm{LR}}$ is the long-range HF exchange integral used in the range-separated functional, written in terms of the $\mathrm{erf}\left(\omega r_{ij}\right)/r_{ij}$ operator (ω and β define the used range-separated functional [93,94,95]). $\varepsilon_{c}$ and $\varepsilon_{x}$ are instead the exchange and correlation energy densities per unit particle, respectively. It is worth remarking that most DFT functionals do not include an explicit description of dispersion interactions. These are generally accounted for by means of empirical corrections, which depend on the atomic positions only, and correct the total energy by an additional parametric term $E^{disp}$. As an alternative, in this work, we consider nonlocal (NL) corrections to the correlation energy $E_{c}$ in Equation (2) [96,97]. Here, NL correlation (NLC) corrections are written in terms of NL VV10 functionals, which correct the $E_{c}$ in Equation (2) by [98]

(3) $$E_{NLC}^{VV10}\left[\rho^{P},\rho^{Q}\right]=\int \rho^{P}\left(r\right)\left(\frac{1}{32}{\left(\frac{3}{b^{2}}\right)}^{3/4}+\frac{1}{2}\int \rho^{Q}\left(r^{\prime }\right)\Phi \left(r,r^{\prime },b,C\right)\;dr^{\prime }\right)\;dr$$

where Φ is the NLC kernel defined in Ref. [96], and P,Q denote two generic fragments (vide infra). The functional depends on the parameter *b*, which controls the short-range damping of the $1/r^{6}$ asymptotic behavior, and on the coefficient *C*, which determines the accuracy of the asymptotic $C_{6}$. In the following, we adopt a more compact notation by grouping Equation (2) into $E_{xc}\left[D\right]$, which, when a nonlocal correlation functional is employed, also includes the $E_{NLC}^{VV10}$ contribution within the correlation term.

For a system composed of two fragments *A* and *B*, KS-FLMOs can be computed by dissecting the total DFT energy into the fragment energies and their interaction energy:

(4a) $$\begin{array}{cc} E & =E_{\left(AB\right)}^{A}+E_{\left(AB\right)}^{B}+E_{int,(AB)}^{AB} \end{array}$$

(4b) $$\begin{array}{cc} E_{\left(AB\right)}^{A} & =\mathrm{Tr}\;h^{A}D^{A}+\frac{1}{2}\mathrm{Tr}\;D^{A}J\left(D^{A}\right)-\frac{1}{2}c_{x}\mathrm{Tr}\;D^{A}K\left(D^{A}\right)+E_{xc}\left[D^{A}\right]+h_{nuc}^{A} \end{array}$$

(4c) $$E_{\left(AB\right)}^{B}=\mathrm{Tr}\;h^{B}D^{B}+\frac{1}{2}\mathrm{Tr}\;D^{B}J\left(D^{B}\right)-\frac{1}{2}c_{x}\mathrm{Tr}\;D^{B}K\left(D^{B}\right)+E_{xc}\left[D^{B}\right]+h_{nuc}^{B}$$

(4d) $$\begin{array}{cc} E_{int,(AB)}^{AB}= & \mathrm{Tr}\;V^{A}D^{B}+\mathrm{Tr}\;V^{B}D^{A}+\mathrm{Tr}\;D^{A}J\left(D^{B}\right)-c_{x}\mathrm{Tr}\;D^{A}K\left(D^{B}\right)-\beta \mathrm{Tr}\;D^{A}K^{\mathrm{LR}}\left(D^{B}\right)\\ & +\int \rho^{A}\left(r\right)\varepsilon_{xc}\left(\rho^{B}\left(r\right)\right)\;dr+\int \rho^{B}\left(r\right)\varepsilon_{xc}\left(\rho^{A}\left(r\right)\right)\;dr+E_{NLC}^{VV10}\left[\rho^{A},\rho^{B}\right]+E_{NLC}^{VV10}\left[\rho^{B},\rho^{A}\right]\\ & +E_{\mathrm{non}-\mathrm{add}}^{AB}+h_{nuc}^{AB} \end{array}$$

where $\rho^{X}\left(r\right),\left(X=\left\{A,B\right\}\right)$ is the fragment density function connected to the fragment density matrix $D^{X}$, which is computed by a Cholesky decomposition from the total density matrix $D=D^{A}+D^{B}$. $\varepsilon_{xc}$ is the exchange-correlation functional, while $h_{nuc}^{A}$ and $h_{nuc}^{B}$ are the nuclear repulsions of the A and B fragments, and $h_{nuc}^{AB}$ is the nuclear repulsion between the A and B nuclei. Finally, $E_{\mathrm{non}-\mathrm{add}}^{AB}$ accounts for the non-linearity of $\varepsilon_{x}$ and $\varepsilon_{c}$ energy functionals per unit particle (see Refs. [69,99,100] for its definition). In Equation (4a), we have introduced the (AB) pedix, which indicates that all the energetic components are calculated by considering the fragment densities that lie in their electronic minimum within the dimer electronic structure. KS-FLMOs are obtained by minimizing the sum of the fragment energies ($E^{A}+E^{B}$) in the space defined by the occupied MOs. In this way, the interaction energy ($E_{int}^{AB}$) and therefore their repulsion are maximized while the total energy *E* is kept constant [70,71,101]. The resulting KS-FLMOs are therefore confined to the pre-defined A and B spatial regions. We refer the interested reader to Ref. [69] for a detailed discussion on the computational protocol to obtain them.

### 2.2. Kohn–Sham Fragment Energy Decomposition Analysis

In this section, we briefly recall the basis of Kohn–Sham Fragment EDA (KS-FEDA), which is an energy decomposition analysis based on KS-FLMOs. The total interaction energy $E^{int}$ reads [69]

(5) $$\begin{array}{cc} E^{int} & =E-E_{\left(0\right)}^{A}-E_{\left(0\right)}^{B}\\ \; & =E_{int,(AB)}^{AB}+E_{\left(AB\right)}^{A}-E_{\left(0\right)}^{A}+E_{\left(AB\right)}^{B}-E_{\left(0\right)}^{B} \end{array}$$

where $E_{\left(0\right)}^{A}$ and $E_{\left(0\right)}^{B}$ are the energies of isolated A and B fragments in the gas phase calculated in the full AO basis set to reduce the basis set superposition error (BSSE) through the counterpoise correction proposed by Boys and Bernard [102]. In Equation (5), *E* is the DFT energy of the full system.

The KS-FEDA approach decomposes the interaction energies of two interacting fragments *A* and *B* into physically meaningful energy components. The KS-FEDA energy terms are obtained by following the following computational protocol:

1. Calculation of A and B DFT energies ($E_{0}^{A}$ and $E_{0}^{B}$) in the gas phase using the full AO basis. The Self-Consistent Field (SCF) converged density matrix of the two fragments is defined as $D_{0}^{A}$ and $D_{0}^{B}$.
2. From the isolated fragment densities, the interaction frozen energy terms can be calculated by [69]
  (6a) $$\begin{array}{cc} E_{\left(0\right)}^{int} & =E_{\left(0\right)}^{ele}+E_{\left(0\right)}^{HF,x}+E_{\left(0\right)}^{corr} \end{array}$$
  (6b) $$\begin{array}{cc} E_{\left(0\right)}^{ele} & =\mathrm{Tr}\;D_{0}^{A}J\left(D_{0}^{B}\right)+\mathrm{Tr}\;V^{A}D_{0}^{B}+\mathrm{Tr}\;V^{B}D_{0}^{A}+h_{\mathrm{rep}}^{AB} \end{array}$$
  (6c) $$\begin{array}{cc} E_{\left(0\right)}^{HF,x} & =-\mathrm{Tr}\;D_{0}^{A}K\left(D_{0}^{B}\right) \end{array}$$
  (6d) $$\begin{array}{cc} E_{\left(0\right)}^{corr} & =E_{xc}\left[D_{0}\right]-E_{xc}\left[D_{0}^{A}\right]-E_{xc}\left[D_{0}^{B}\right]-\left(1-c_{x}\right)E_{\left(0\right)}^{HF,x} \end{array}$$
  where $D_{0}=D_{0}^{A}+D_{0}^{B}$. $E_{\left(0\right)}^{ele}$, $E_{\left(0\right)}^{HF,x}$, and $E_{\left(0\right)}^{corr}$ represent the electrostatic, HF exchange, and correlation energy contributions to the frozen interaction energy.
3. To obtain a physically sound description, the antisymmetry of the total wavefunction must be enforced. A convenient route is to orthonormalize the dimer MOs (i.e., $\phi_{0}=\phi_{0}^{A}⊕\phi_{0}^{B}$) using a Löwdin procedure [103]. From the orthonormalized MOs, the density matrix $D_{\mathrm{ASN}}$ and density function $\rho^{\mathrm{ASN}}$ related to the antisymmetrized (ASN) wavefunction can be calculated. These define the physically admissible single-determinant representation of the frozen fragment MOs. The associated energy $E^{\mathrm{ASN}}$ is then evaluated by inserting $D_{\mathrm{ASN}}$ and $\rho_{\mathrm{ASN}}$ into Equation (1).
4. By antisymmetrizing the system, we obtain a delocalized wavefunction. Therefore, a partial charge transfer between the two fragments occurs. We can then perform a KS-FLMO procedure to construct the density matrices ($D_{\mathrm{ASN}}^{A},D_{\mathrm{ASN}}^{B}$) and energies ($E_{\mathrm{ASN}}^{A},E_{\mathrm{ASN}}^{B}$) of the moieties in the electronic structure of the ASN dimer. This allows us to decompose the ASN energy $E^{\mathrm{ASN}}$ into the electrostatic, HF exchange, correlation, and electronic preparation energies as [69]
  (7a) $$\begin{array}{cc} E_{\mathrm{ASN}}^{int} & =E_{\mathrm{ASN}}^{ele}+E_{\mathrm{ASN}}^{HF,x}+E_{\mathrm{ASN}}^{corr}+E_{\mathrm{ASN}}^{el-prep} \end{array}$$
  (7b) $$\begin{array}{cc} E_{\mathrm{ASN}}^{ele} & =\mathrm{Tr}\;D_{\mathrm{ASN}}^{A}J\left(D_{\mathrm{ASN}}^{B}\right)+\mathrm{Tr}\;\;V^{A}D_{\mathrm{ASN}}^{B}+\mathrm{Tr}\;\;V^{B}D_{\mathrm{ASN}}^{A}+h_{\mathrm{rep}}^{AB} \end{array}$$
  (7c) $$\begin{array}{cc} E_{\mathrm{ASN}}^{HF,x} & =-\mathrm{Tr}\;D_{\mathrm{ASN}}^{A}K\left(D_{\mathrm{ASN}}^{B}\right) \end{array}$$
  (7d) $$\begin{array}{cc} E_{\mathrm{ASN}}^{corr} & =E_{xc}\left[D_{\mathrm{ASN}}\right]-E_{xc}\left[D_{\mathrm{ASN}}^{A}\right]-E_{xc}\left[D_{\mathrm{ASN}}^{B}\right]-\left(1-c_{x}\right)E_{\mathrm{ASN}}^{HF,x} \end{array}$$
  (7e) $$\begin{array}{cc} E_{\mathrm{ASN}}^{el-prep} & =E_{\mathrm{ASN}}^{A}-E_{\left(0\right)}^{A}+E_{\mathrm{ASN}}^{B}-E_{\left(0\right)}^{B} \end{array}$$
  We can now calculate the energy change due to the antisymmetrization of the total wavefunction, $\Delta E_{\mathrm{ASN}}$, which can be further decomposed into electrostatic, HF exchange, and correlation contributions:
  (8a) $$\begin{array}{cc} \Delta E_{\mathrm{ASN}} & =\Delta E_{\mathrm{ASN}}^{ele}+\Delta E_{\mathrm{ASN}}^{HF,x}+E_{\mathrm{ASN}}^{el-prep}+\Delta E_{\mathrm{ASN}}^{corr} \end{array}$$
  (8b) $$\begin{array}{cc} \; & =\Delta E_{\mathrm{ASN}}^{HF}+\Delta E_{\mathrm{ASN}}^{corr} \end{array}$$
  (8c) $$\begin{array}{cc} \Delta E_{\mathrm{ASN}}^{ele} & =E_{\mathrm{ASN}}^{ele}-E_{\left(0\right)}^{ele} \end{array}$$
  (8d) $$\begin{array}{cc} \Delta E_{\mathrm{ASN}}^{HF,x} & =E_{\mathrm{ASN}}^{HF,x}-E_{\left(0\right)}^{HF,x} \end{array}$$
  (8e) $$\begin{array}{cc} \Delta E_{\mathrm{ASN}}^{corr} & =E_{\mathrm{ASN}}^{corr}-E_{\left(0\right)}^{corr} \end{array}$$
  where $\Delta E_{\mathrm{ASN}}^{HF}$ contains the energy variations due to electrostatic, HF exchange, and electronic preparation. By combining Equation (8) with Equation (7a), we can define the electrostatic ($E^{ele}=E_{\left(0\right)}^{ele}$), exchange ($E^{ex}=E_{\left(0\right)}^{HF,x}$), and repulsion ($E^{rep}=\Delta E_{\mathrm{ASN}}^{HF}$) quantities that are commonly exploited in other EDA techniques:
  (9) $$E_{\mathrm{ASN}}^{int}=E^{ele}+E^{ex}+E^{rep}+E_{\mathrm{ASN}}^{corr}$$
5. To obtain the energetic contributions due to orbital relaxation, the total system DFT energy *E* is minimized (see Equation (1)).
6. The Self-Consistent Field (SCF)-converged density of the total system fully accounts for charge transfer between the two subsystems and the orbital relaxation. The obtained MOs are localized by an additional KS-FLMO procedure, to obtain the fragment density matrices ($D_{\left(AB\right)}^{A},D_{\left(AB\right)}^{B}$) and energies ($E_{\left(AB\right)}^{A},E_{\left(AB\right)}^{B}$) in the relaxed electronic structure of the dimer.
7. By using the definition of $E_{int,(AB)}^{AB}$ (see Equation (4d), readjusted in a GKS framework; see Equation (1b)), the total interaction energy (Equation (5)) can be finally decomposed as [69]
  (10a) $$\begin{array}{cc} E^{int} & =E_{\left(AB\right)}^{ele}+E_{\left(AB\right)}^{HF,x}+E_{\left(AB\right)}^{corr}+E_{\left(AB\right)}^{el-prep}+E^{disp} \end{array}$$
  (10b) $$\begin{array}{cc} E_{\left(AB\right)}^{ele} & =\mathrm{Tr}\;D_{\left(AB\right)}^{A}J\left(D_{\left(AB\right)}^{B}\right)+\mathrm{Tr}\;\;V^{A}D_{\left(AB\right)}^{B}+\mathrm{Tr}\;\;V^{B}D_{\left(AB\right)}^{A}+h_{\mathrm{rep}}^{AB} \end{array}$$
  (10c) $$\begin{array}{cc} E_{\left(AB\right)}^{HF,x} & =-\mathrm{Tr}\;D_{\left(AB\right)}^{A}K\left(D_{\left(AB\right)}^{B}\right) \end{array}$$
  (10d) $$\begin{array}{cc} E_{\left(AB\right)}^{corr} & =E_{xc}\left[D\right]-E_{xc}\left[D_{\left(AB\right)}^{A}\right]-E_{xc}\left[D_{\left(AB\right)}^{B}\right]-\left(1-c_{x}\right)E_{\left(AB\right)}^{HF,x} \end{array}$$
  (10e) $$\begin{array}{cc} E_{\left(AB\right)}^{el-prep} & =E_{\left(AB\right)}^{A}-E_{\left(0\right)}^{A}+E_{\left(AB\right)}^{B}-E_{\left(0\right)}^{B} \end{array}$$
  (10f) $$\begin{array}{cc} E^{disp} & =E_{AB}^{disp}-E_{A}^{disp}-E_{B}^{disp} \end{array}$$
  where $E^{disp}$ accounts for the dispersion interaction in additive schemes (such as Grimme’s D3 or D4 [81,82,83,84,85,86]) and is calculated from the dispersion energies of the A and B monomers ($E_{A}^{disp}$, $E_{B}^{disp}$), and the dimer ($E_{AB}^{disp}$). We remark that, in case an NLC functional is exploited, this term is accounted for by the correlation energy contribution. In both cases, it is worth highlighting that KS-FEDA total interaction energies $E^{int}$ correspond to the interaction energies computed using the underlying density functional [69]. In general, interaction energies computed using KS-FEDA display energy deviations with respect to CCSD(T) well below the chemical accuracy (1 kcal/mol) for common intermolecular datasets (A24 [104], S22 [105], and IHB15 [106]).
  By combining Equations (9) and (10), we can decompose the total interaction energy (see Equation (5)) as follows:
  (11) $$E^{int}=E^{ele}+E^{ex}+E^{rep}+E_{ASN}^{corr}+\Delta E_{\mathrm{orb}}+E^{disp}$$
  where $\Delta E_{\mathrm{orb}}$ accounts for the interaction energy due to the orbital relaxation, and reads
  (12a) $$\begin{array}{cc} \Delta E_{\mathrm{orb}} & =\Delta E_{\mathrm{orb}}^{ele}+\Delta E_{\mathrm{orb}}^{HF,x}+\Delta E_{\mathrm{orb}}^{el-prep}+\Delta E_{\mathrm{orb}}^{corr}\\ & =\Delta E_{\mathrm{orb}}^{HF}+\Delta E_{\mathrm{orb}}^{corr} \end{array}$$
  (12b) $$\begin{array}{cc} \Delta E_{\mathrm{orb}}^{ele} & =E_{\left(AB\right)}^{ele}-E_{\mathrm{ASN}}^{ele} \end{array}$$
  (12c) $$\begin{array}{cc} \Delta E_{\mathrm{orb}}^{HF,x} & =E_{\left(AB\right)}^{HF,x}-E_{\mathrm{ASN}}^{HF,x} \end{array}$$
  (12d) $$\begin{array}{cc} \Delta E_{\mathrm{orb}}^{el-prep} & =E_{\left(AB\right)}^{el-prep}-E_{\mathrm{ASN}}^{el-prep} \end{array}$$
  (12e) $$\begin{array}{cc} \Delta E_{\mathrm{orb}}^{corr} & =E_{\left(AB\right)}^{corr}-E_{\mathrm{ASN}}^{corr} \end{array}$$
  By explicating all the terms in Equation (11) (using Equations (8) and (12)), we obtain the total 12-term KS-FEDA [69]:
  (13) $$\begin{array}{cc} E^{int} & =E^{ele}+E^{ex}+E_{\left(0\right)}^{corr}\\ \; & \;+\Delta E_{\mathrm{ASN}}^{ele}+\Delta E_{\mathrm{ASN}}^{HF,x}+E_{\mathrm{ASN}}^{el-prep}+\Delta E_{\mathrm{ASN}}^{corr}\\ \; & \;+\Delta E_{\mathrm{orb}}^{ele}+\Delta E_{\mathrm{orb}}^{HF,x}+\Delta E_{\mathrm{orb}}^{el-prep}+\Delta E_{\mathrm{orb}}^{corr}\\ \; & \;+E^{disp} \end{array}$$
  This energy decomposition allows for investigating the physical origin of the $E^{rep}=\Delta E_{\mathrm{ASN}}^{HF}$ and $\Delta E_{\mathrm{orb}}^{HF}$ energy terms, and, remarkably, underscores how the interaction energy terms evolve as the densities of the monomers evolve as the adduct is electronically formed. Furthermore, Equation (11) can also be rewritten by including the total correlation energy $E_{\left(AB\right)}^{corr}=E^{corr}$ (see Equation (10d)), resulting in a six-term KS-FEDA:
  (14) $$E^{int}=E^{ele}+E^{ex}+E^{rep}+E^{corr}+\Delta E_{\mathrm{orb}}^{HF}+E^{disp}$$
  where $E^{ex}$ and $E^{rep}$ can also be combined, similarly to most EDA schemes, such as KM-EDA and SAPT. In particular, SAPT decomposes the total interaction energy into electrostatic, exchange (also accounting for repulsive effects), polarization (or induction), and dispersion. We have recently shown that KS-FEDA energy terms directly correlate with SAPT ones, by the following correspondence [69]
  (15a) $$\begin{array}{cc} E^{ele} & :E_{\mathrm{SAPT}}^{ele}↔E_{\mathrm{KS}-\mathrm{FEDA}}^{ele} \end{array}$$
  (15b) $$\begin{array}{cc} E^{ex-rep} & :E_{\mathrm{SAPT}}^{exch}↔E_{\mathrm{KS}-\mathrm{FEDA}}^{ele} \end{array}$$
  (15c) $$\begin{array}{cc} E^{pol} & :E_{\mathrm{SAPT}}^{ind}↔\Delta E_{\mathrm{orb},\mathrm{KS}-\mathrm{FEDA}}^{HF} \end{array}$$
  (15d) $$\begin{array}{cc} E^{disp} & :E_{\mathrm{SAPT}}^{disp}↔\Delta E_{\mathrm{KS}-\mathrm{FEDA}}^{corr}+E^{disp} \end{array}$$
  To summarize, the KS-FEDA protocol comprises (i) two monomer SCF optimizations in the full AO basis, (ii) two KS-FLMO localization cycles, and (iii) one SCF on the dimer.

## 3. Results

**Figure 1:**
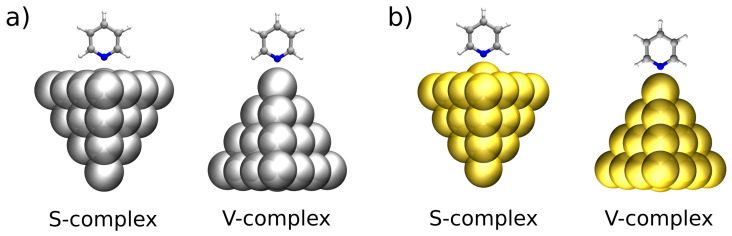
Graphical representation of pyridine adsorbed on tetrahedral Ag_20_ (**a**) and Au_20_ (**b**) nanoparticles in the S (surface) and V (vertex) configurations.

KS-FEDA is applied to analyze the energetic components driving the interaction between pyridine and noble metal nanoparticles (NPs) composed of gold (Py-Au) and silver (Py-Ag). In particular, we employ structural models that have previously been fully optimized and characterized in Refs. [87,89], where pyridine is adsorbed on small Ag and Au NPs of 20 atoms (Ag_20_ and Au_20_). These are tetrahedral clusters that can be viewed as relaxed fragments of the face-centered cubic (fcc) lattice of the corresponding bulk metals [107,108]. The tetrahedral cluster is one of the local minima of Ag_20_ at the DFT level [107,108], and is reported to be the most stable for Au_20_ [109]. These NPs offer two distinct adsorption sites that differ in the local coordination and electronic environment. In the first morphology, pyridine is adsorbed on top of one of the four (111) facets of the tetrahedron, mimicking adsorption on an extended metallic surface (S-complex). Alternatively, the molecule coordinates to one of the cluster vertices (V-complex), representing an adatom-like site. In both configurations, the interaction occurs through the nitrogen atom of pyridine, oriented perpendicularly to the surface (or vertex). A graphical depiction of Py-Ag and Py-Au in both S- and V-complexes is given in Figure 1.

The equilibrium metal–nitrogen distances reflect the diverse coordination environments: for Py-Ag_20_, they are 2.66 Å (S-complex) and 2.46 Å (V-complex), while for Py-Au_20_, they shorten to 2.33 Å and 2.23 Å. This contraction for gold is consistent with the stronger stabilization and enhanced polarizability of Au compared to Ag, which is also characterized by a distortion of the closest Au atom in the S-complex (see Figure 1b). In the following, two density functionals are considered: the range-separated hybrid CAM-B3LYP [94] corrected with empirical dispersion D4 [84,85,86], and NLC SCAN-rVV10 [98], which has been reported to provide accurate interaction energies for molecular systems interacting with coinage metal surfaces [98]. All the KS-FEDA calculations are performed with a developed version of the electronic structure software e^*T*^ 1.0 [110].

### 3.1. Basis Set Dependence

**Figure 2:**
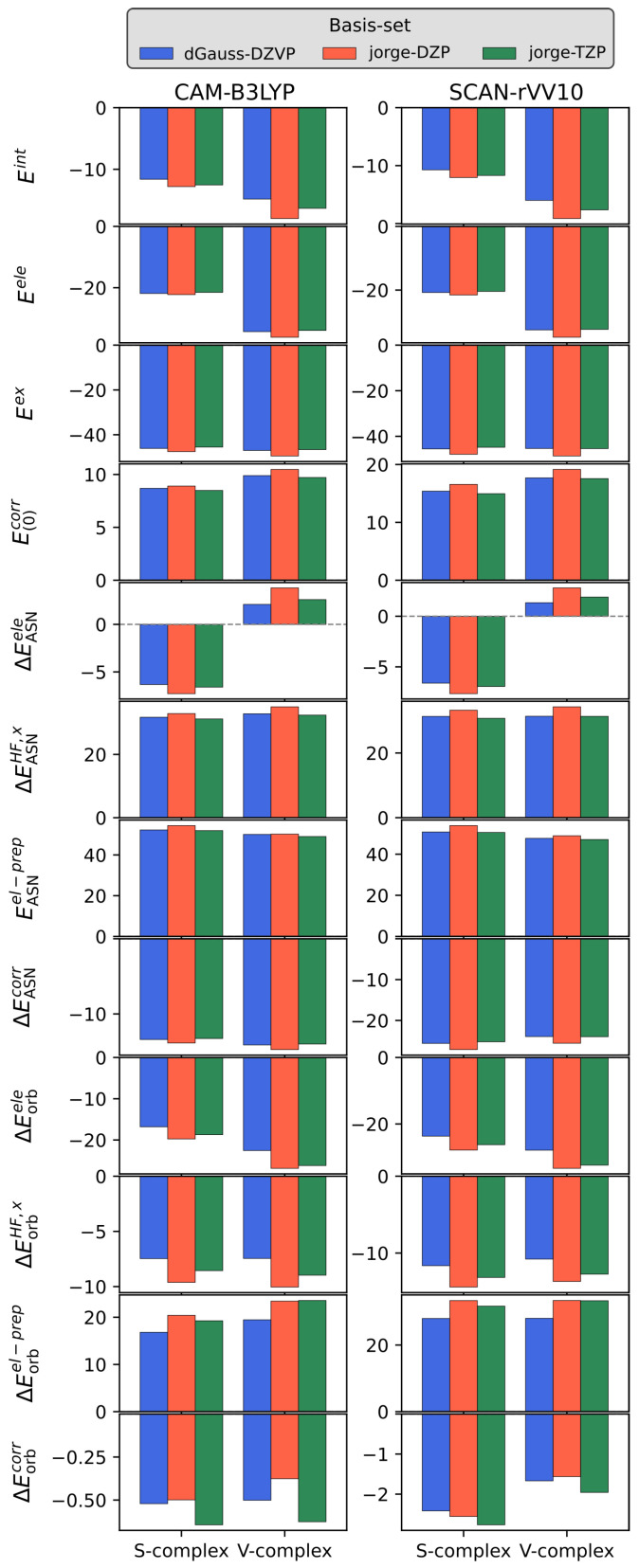
KS-FEDA energy terms (see Equation (13)) for Py-Ag S- and V-complex by exploiting CAM-B3LYP and SCAN-rVV10 combined with dGauss-DZVP, jorge-DZP, and jorge-TZP basis sets. All energy terms are given in kcal/mol.

We start our discussion by analyzing the basis set dependence of the full KS-FEDA decomposition (see Equation (13)). In particular, we compute the KS-FEDA for both S- and V-complexes of the Py-Ag adduct by exploiting CAM-B3LYP and SCAN-rVV10. In both cases, all-electron basis sets with double- (DZ) and triple-zeta (TZ) quality and polarization functions are used: dGauss-DZVP [111,112], jorge-DZP [112,113], and jorge-TZP [112], which is taken as a reference. The results are graphically depicted in Figure 2 (raw data reported in Tables S1–S3 in the Supplementary Materials).

For the S-complex, CAM-B3LYP yields a moderately attractive total interaction energy (computed by including the dispersion correction at the D4 level) that is only weakly basis-dependent: $E^{int}=-11.61$, −12.81, and −12.54 kcal/mol with dGauss-DZVP, jorge-DZP, and jorge-TZP, respectively. By taking jorge-TZP as a reference, the deviations are rather small and below the chemical accuracy (1 kcal/mol) for both DZ basis sets. By looking to the specific energy contribution, electrostatics and exchange are both attractive, and only slight deviations are reported with respect to jorge-TZP values. $E_{\left(0\right)}^{corr}$ is moderately destabilizing (8.5–8.9 kcal/mol), similarly to what has been reported previously for molecular systems [69]. Relative to jorge-TZP, the deviations in the frozen interaction energy terms are all below the chemical accuracy, except for $E^{ex}$ (jorge-DZP), for which, however, a very small relative error of about 4.5% is reported.

Within KS-FEDA, we can also compute the energy variation by antisymmetrizing the frozen fragment densities, resulting in ASN terms. In this case, $\Delta E_{\mathrm{ASN}}^{ele}$ and $\Delta E_{\mathrm{ASN}}^{HF,x}$ are characterized by an opposite behavior, being attractive and repulsive, respectively. The deviations with respect to jorge-TZP are particularly small for $\Delta E_{\mathrm{ASN}}^{ele}$ (0.27 and 0.69 kcal/mol). For $\Delta E_{\mathrm{ASN}}^{HF,x}$, the deviations are 0.53 (dGauss-DZVP) and 1.70 kcal/mol for jorge-DZP, which, however, represents only a relative error of about 5%. A relevant electronic-preparation cost $E_{\mathrm{ASN}}^{el-prep}\approx$ 52–54 kcal/mol is reported, underlying a substantial electronic reorganization to obtain a correct physical state from the two isolated fragments. Also in this case, for jorge-DZP, we report an error above the chemical accuracy (2.45 kcal/mol), which is, however, associated again with a very small relative error (4.7%). The electronic preparation term is partly compensated for by $\Delta E_{\mathrm{ASN}}^{corr}$ values which are between −13.3 and −13.9 kcal/mol, and whose deviations remain within 1 kcal/mol (0.13 and 0.60) for both DZ basis sets. Orbital relaxation effects further stabilize the complex via $\Delta E_{\mathrm{orb}}^{ele}$ (ranging between −16.8 and −19.8 kcal/mol) and $\Delta E_{\mathrm{orb}}^{HF,x}$ (ranging between −7.5 and −9.6 kcal/mol), which are however counterbalanced by the repulsive $\Delta E_{\mathrm{orb}}^{el-prep}$ (ranging between 16.8 and 20.4 kcal/mol). A small but favorable $\Delta E_{\mathrm{orb}}^{corr}$ (about −0.5/−0.6 kcal/mol) is also reported for all basis sets. For orbital-relaxation corrections, dGauss-DZVP provides the worst agreement with the reference jorge-TZP values, also showing the maximum absolute error (about 13 %).

By exploiting the SCAN-rVV10 functional, the total interaction energy is consistently predicted as attractive, $E^{int}=-10.73$, −12.06, and −11.70 kcal/mol, and relative to jorge-TZP, the deviations are 0.97 (dGauss-DZVP) and 0.36 (jorge-DZP) kcal/mol, which are in both cases below the chemical accuracy. By comparing the functionals by keeping fixed the basis set, SCAN-rVV10 weakens the non-covalent interaction by 0.88 (dGauss-DZVP), 0.75 (jorge-DZP), and 0.84 (jorge-TZP) kcal/mol with respect to CAM-B3LYP-D4. The frozen electrostatics/exchange remain of similar magnitude to CAM-B3LYP, but $E_{\left(0\right)}^{corr}$ is noticeably more repulsive (15.0–16.6 kcal/mol). This is expected because the correlation energy term in the NLC SCAN-rVV10 functional also accounts for dispersion interactions. Deviations of $E_{\left(0\right)}^{corr}$ versus the SCAN-rVV10 jorge-TZP reference amount to 0.44 (dGauss-DZVP) and 1.61 (jorge-DZP) kcal/mol (about 10.8%). KS-FEDA reveals a larger symmetry adaptation energy under SCAN-rVV10: the ASN correlation is markedly more stabilizing, ranging from −25 and −27 kcal/mol. This is again due to the accounting of dispersion interactions. Also, in this case, the maximum discrepancy between the methods is rather small and below the chemical accuracy. In general, orbital relaxation terms result in a stabilization of the adduct, with $\Delta E_{\mathrm{orb}}^{ele}$ ranging from −23.7 to −27.9, and $\Delta E_{\mathrm{orb}}^{HF,x}$ ranging from 11.7 to −14.4. However, a large electronic preparation penalty ($\Delta E_{\mathrm{orb}}^{el-prep}$, ranging from 28.0 to 33.3 kcal/mol) is also reported, which overall makes the results from SCAN-rVV10 close to CAM-B3LYP-D4. For SCAN-rVV10, however, we highlight a discrepancy between dGauss-DZVP and jorge-DZP/jorge-TZP for the orbital relaxation contributions, with comparatively large deviations (about 3.61 kcal/mol for $\Delta E_{\mathrm{orb}}^{el-prep}$).

We now move to the V-complex (see Figure 2 and Table S3 in the Supplementary Materials). By exploiting the CAM-B3LYP (with D4 dispersion) functional, the adsorption energy is larger than in the S-complex, and is also associated with a higher basis set sensitivity. The deviation in $E^{int}$ exceeds chemical accuracy for both dGauss-DZVP and jorge-DZP with respect to jorge-TZP. This, however, corresponds to a relatively small absolute error of about 9–10%. In contrast, the frozen energy contributions are very similar upon varying the basis set: for dGauss-DZVP, the deviations in $E^{ele}$ and $E^{ex}$ are within chemical accuracy, while for jorge–DZP, they are modestly larger but with an absolute error of about 6–7%. The frozen correlation $E_{\left(0\right)}^{corr}$ is also destabilizing for the V-complex but, for both basis sets, is close to convergence, with very small differences (<1 kcal/mol) against the TZ reference.

The symmetry-adapted (ASN) energy terms display a specific repulsive pattern for both $\Delta E_{\mathrm{ASN}}^{ele}$ and $\Delta E_{\mathrm{ASN}}^{HF,x}$, different from S-complex and other molecular systems previously investigated at the KS-FEDA level [69]. All ASN terms differ from the jorge-TZP reference by less than 1 kcal/mol. The only notable exception is $\Delta E_{\mathrm{ASN}}^{HF,x}$ at jorge-DZP, which exceeds chemical accuracy, but again shows a moderate relative error of about 8%. The ASN electronic-preparation term $E_{\mathrm{ASN}}^{el-prep}$ is large, as expected for a strongly symmetry-adapted state, but it is essentially converged across the basis sets, and the ASN correlation $\Delta E_{\mathrm{ASN}}^{corr}$ provides a consistent stabilization that also remains within chemical accuracy for all basis sets. The basis set sensitivity is dominant in the orbital-relaxation energy contributions, which also provides the overall stabilization of the dimer. With jorge-DZP, all these terms track the TZ reference within chemical accuracy. In contrast, dGauss-DZVP shows deviations that are more substantial and exceed chemical accuracy for the most sensitive orbital components. Remarkably, these also correspond to more substantial absolute errors ranging from 14 to 17%.

By exploiting the NLC SCAN-rVV10 functional, adsorption becomes more attractive by roughly 1–1.5 kcal/mol, but the overall qualitative picture discussed above remains valid. The total interaction energy again is slightly above the chemical accuracy for dGauss-DZVP and jorge-DZP when compared with jorge-TZP, corresponding to an error of 8–9%. The frozen electrostatics and exchange are essentially converged for dGauss-DZVP (within chemical accuracy) and remain moderate for jorge-DZP (about 8–10%). As expected for SCAN-rVV10 due to the inclusion of nonlocal terms, $E_{\left(0\right)}^{corr}$ is more repulsive. However, the deviation stays within chemical accuracy for dGauss-DZVP and is modestly above it for jorge-DZP, which, however, provides an absolute error of about 9%. Most ASN energy contributions remain within chemical accuracy, except for $\Delta E_{\mathrm{ASN}}^{HF,x}$ at jorge-DZP (2.50 kcal/mol, error 9%). The ASN preparation energy increases slightly with respect to CAM-B3LYP, but for both DZ basis sets, it is close to convergence. For the orbital-relaxation energy terms, jorge-DZP once more reproduces the TZP picture within chemical accuracy for all terms, whereas dGauss-DZVP exhibits substantial discrepancies that correspond to ∼14–16% absolute errors.

The KS-FEDA results can also be compacted into SAPT-like components (electrostatics, induction/polarization, dispersion, and exchange–repulsion) to enable a direct comparison with state-of-the-art methods (see Equation (15)). Also, in this case, the absolute discrepancies among the individual components when moving from jorge-TZP to smaller bases are generally close to chemical accuracy. In particular, it is worth remarking that, although some discrepancies from this trend do occur, the underlying physical picture remains unchanged for both S- and V-complexes. In fact, the distribution of the interaction energy across the various components is preserved: taking the exchange–repulsion, $E^{ex-rep}$, as an internal reference, the relative percentage balance remains almost identical across basis sets for both CAM-B3LYP and SCAN-rVV10 (see Tables S2–S4 in the Supplementary Materials). Indeed, the various basis sets predominantly rescale the component magnitudes slightly, most noticeably in the ex-rep and induction/polarization terms, without modifying their hierarchy that delivers the final $E^{int}$. As a consequence, the physicochemical aspects of the binding remain correctly captured by using economical basis sets. Therefore, in the following analysis, we exploit the jorge-DZP basis set, because it provides a good compromise between accuracy and computational cost, also allowing calculations on larger, electron-richer metal substrates, such as gold.

### 3.2. KS-FEDA on Py-Ag and Py-Au

**Figure 3:**
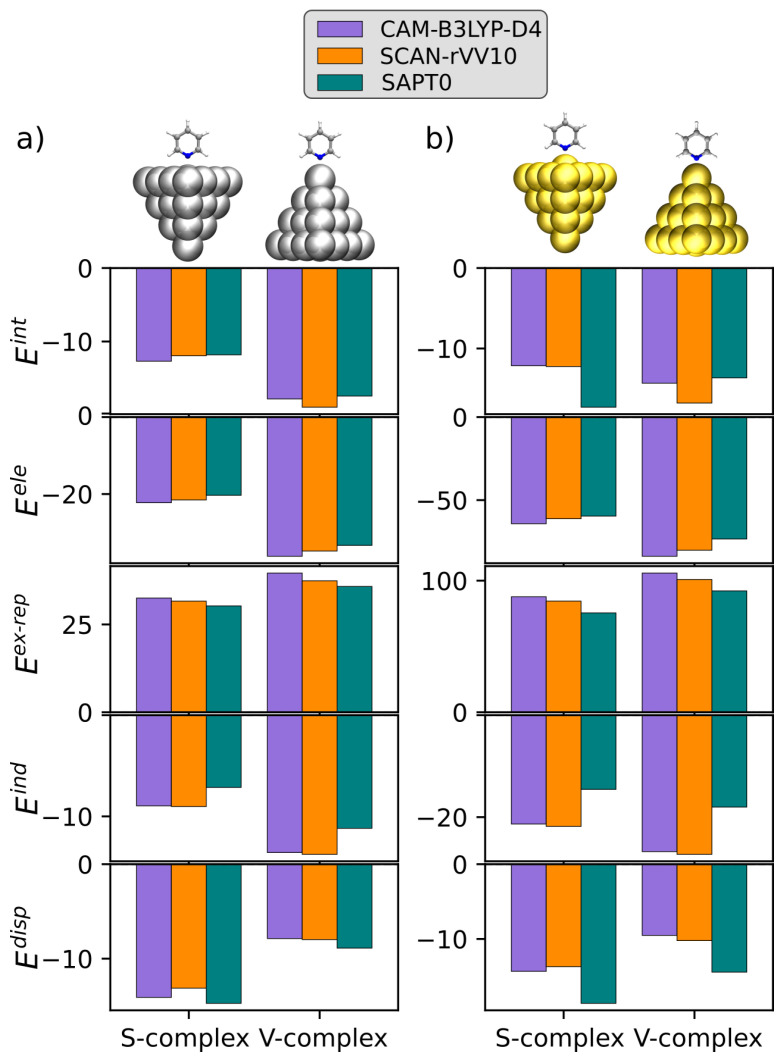
KS-FEDA energy terms (Equation (15)) for Py-Ag (**a**) and Py-Au (**b**) S- and V-complexes at the CAM-B3LYP-D4 and SCAN-rVV10 levels. SAPT0 results are also reported for comparison. In all cases, the jorge-DZP basis set is used. All energy terms are given in kcal/mol.

We now move to comment on the KS-FEDA for both Py-Ag and Py-Au complexes in surface and vertex adsorption sites. To this end, we compare KS-FEDA based on CAM-B3LYP-D4 and SCAN-rVV10, to Symmetry Adapted Perturbation Theory SAPT0 [54] by exploiting the jorge-DZP basis set. To this end, we exploit the minimal KS-FEDA decomposition in Equation (15). SAPT0 calculations are performed using Psi4 1.9 [114,115]. All the results are graphically depicted in Figure 3 and also reported in Table 1.

By first focusing on Py-Ag (S-complex), we note that CAM-B3LYP–D4 and SCAN–rVV10 yield similar total interaction energies, with SCAN-rVV10 predicting a slightly weaker binding than CAM-B3LYP-D4, while SAPT0 is only marginally less attractive than CAM-B3LYP and essentially matches SCAN-rVV10 at the level of the total energy. Remarkably, the three methods predict very close total interaction energies, with differences that are below the chemical accuracy. By examining the specific energy components, we observe that the energy distribution remains stable across the various methods. In fact, $E^{ex-rep}$ is the largest term in absolute value, and $E^{ele}$ is the second contribution (about 60% with respect to the ex-rep term in absolute value). The polarization energy term $E^{pol}$ instead differs between KS-FEDA and SAPT0. While it is comparable in magnitude for CAM-B3LYP and SCAN-rVV10, it reduces by almost 2 kcal/mol in SAPT0. Differently, $E^{disp}$ is the third energy component for all methods. We note that, in SAPT0, a larger fraction of the attraction is distributed to dispersion, and a correspondingly smaller share is attributed to electrostatics/induction. These shifts are small and nearly compensate. In fact, as commented above, the total interaction energy $E^{int}$ remains consistent across the approaches.

For the V-complex, adsorption strengthens relative to the S-complex for all methods by about 5 kcal/mol. In this case, SCAN-rVV10 predicts a more attractive interaction than CAM–B3LYP-D4 (by about 1.1 kcal/mol), while SAPT0 yields very similar results to CAM-B3LYP-D4 (with a discrepancy of just 0.4 kcal/mol). To understand such a discrepancy, energy decomposition analysis is particularly useful. In fact, by moving from CAM-B3LYP to SCAN-rVV10, the repulsive $E^{ex-rep}$ decreases more than the accompanying loss in $E^{ele}$. Also, the polarization and dispersive terms ($E^{pol}$ and $E^{disp}$) are predicted to be very close by the two functionals, tilting the compensation in favor of stronger binding for SCAN-rVV10. Similarly to S-complex, SAPT0 predicts a more attractive $E^{disp}$ and a more repulsive $E^{ele}/E^{pol}$. However, the two effects again tend to compensate, so that the effect on the total interaction energy $E^{int}$ remains small.

Remarkably, the relative weight of the energy components is predicted very similarly by all methods. In fact, the exchange–repulsion energy dominates, electrostatics is the leading attractive term, and induction/dispersion energies provide additional stabilization without altering the overall hierarchy. However, an important difference with S-complex is highlighted: while, for the S-complex, the surface-interaction polarization and dispersion are very close to electrostatics, in the V-complex, the electrostatics is consistently more attractive by almost 10 kcal/mol. Furthermore, as commented above, for the S-complex, the dispersion interaction overcomes the polarization term by about 4–7 kcal/mol. On the contrary, for the V-complex, the opposite is reported. This feature is captured consistently by both KS-FEDA and SAPT0. This can be rationalized by considering that, in the S-complex, pyridine is interacting with a large surface, highlighting a crucial role of dispersion. In V-complex, the interaction is instead atom–atom-like; thus, the electrostatic terms ($E^{ele}$ and $E^{pol}$) dominate the interaction. To quantitatively analyze these terms, in Table 2, we report the relative percentages between each attractive component ($E^{ele}$, $E^{pol}$, and $E^{disp}$) and their sum (see also Table S5 in the Supplementary Materials). The given percentages confirm the trends discussed above. In fact, all methods provide a very close picture of the stabilizing energy terms. Polarization and dispersion terms are particularly relevant (51–52%) for S-complex, while for V-complex, the electrostatics is the most important stabilizing contribution. This is mainly due to the substantial reduction in the dispersion energy terms, which move from 30–35% (S-complex) to 14–17% (V-complex), while polarization remains similar in the two systems.

These aspects can be further appreciated by comparing our EDA results obtained by using the extended transition state method [116,117,118] with the BP86/TZP results reported in the literature [87]. BP86 is a pure GGA functional with no explicit nonlocal dispersion [119,120]. While $E^{ele}$, $E^{ex-rep}$, and $E^{pol}$ are consistent with our findings (with maximum differences of about 3 kcal/mol), the absence of a proper $E^{disp}$ description leads to a severe underestimation of $E^{\mathrm{tot}}$ for both S-complex and V-complex. This highlights that an accurate dispersion treatment, which can be achieved by using DFT+D, NLC functionals, or SAPT, is indeed essential for quantitative adsorption energies.

We now move to the results obtained for the Py-Au complexes (see Figure 3b and Table 3). For the S-complex, we note that CAM-B3LYP-D4 and SCAN-rVV10 yield essentially the same total interaction energy $E^{int}$ with a difference of just 0.1 kcal/mol, whereas SAPT0 predicts a noticeably stronger adsorption with $E^{int}$ more attractive by about 5 kcal/mol. Considering that the Py-Au equilibrium distance is in this case particularly small (2.33 Å), such a discrepancy can be associated with the strong over-binding reported by SAPT0 at short range [54]. The energy distribution is similarly predicted by the three approaches. In all cases, $E^{ex-rep}$ is the largest term in absolute value. $E^{ele}$ is the leading attractive component, while $E^{pol}$ is larger in absolute value than $E^{disp}$ for KS-FEDA, while the opposite is valid for SAPT0. Relative to CAM-B3LYP-D4, SCAN-rVV10 slightly softens both $E^{ex-rep}$ and $E^{ele}$, while leaving $E^{pol}$ comparable and $E^{disp}$ only marginally smaller; these shifts compensate almost entirely when summed up together to $E^{int}$. SAPT0 preserves the same hierarchy but redistributes the attraction by predicting a larger fraction of $E^{disp}$ with respect to $E^{pol}$, and, to a lesser extent, to $E^{ele}$. The overall discrepancy between KS-FEDA and SAPT0 can be ascribed to the cited over-binding by SAPT0 at short range, and electron correlation, which is only partially accounted for in SAPT0. In fact, we remark that KS-FEDA provides an accuracy comparable to that achievable by using high-level golden standard SAPT methods (i.e., SAPT2 + (3)*δ*MP2) at a much lower computational cost [69].

Moving to the V-complex, the adsorption strengthens with respect to the S-complex for all approaches. In particular, SCAN-rVV10 predicts a more attractive $E^{int}$ than CAM-B3LYP-D4 by about 2.5 kcal/mol. SAPT0 instead yields a total interaction energy close to CAM-B3LYP-D4, and is thus less attractive than SCAN-rVV10. The energy decomposition once again helps clarify the origin of the reported trends: when moving from CAM-B3LYP-D4 to SCAN-rVV10, the repulsive $E^{ex-rep}$ decreases more than the accompanying reduction in $E^{ele}$, and, similarly to S-complex and Py-Ag, $E^{pol}$ and $E^{disp}$ are maintained almost unaltered (within the chemical accuracy), resulting in a stronger $E^{int}$ for SCAN-rVV10. SAPT0 again assigns a larger share of attraction to $E^{disp}$ while reducing $E^{ele}$ and especially $E^{pol}$; these shifts tend to cancel out, and the final $E^{int}$ is therefore close to CAM-B3LYP-D4 despite the different partition. As for the S-complex, the relative weights referenced to $E^{ex-rep}$ are predicted very similarly by all approaches: exchange–repulsion dominates, $E^{ele}$ is the leading attractive term, and $E^{pol}/E^{disp}$ contributions provide additional stabilization without altering the overall hierarchy. Compared to the S-complex, the V-complex accentuates the absolute values of electrostatics and induction over dispersion, consistent with a more localized, atom–atom interaction at the vertex site. Different from the Py-Ag adduct, the relative percentages reported in Table 2 (see also Table S7 in the Supplementary Materials) highlight that electrostatics dominates the interaction in both S- and V-complexes. Indeed, we note that, while the relative weight of the polarization term is similar to Py-Ag (between 21 and 23% for KS-FEDA and 16 and 17% for SAPT0), the dispersion interaction percentage decreases but remains substantially larger for the S-complex than the V-complex.

Table 3 also gives the total interaction energies calculated at the BP86/TZP + ZORA reported in the literature [89]. We remark again that BP96 lacks a proper description of $E^{disp}$ interactions. As a result, it substantially underestimates $E^{int}$ for the S-complex. On the contrary, for the V-complex, an apparent error cancellation yields a total $E^{int}$ closer to our results.

We can now compare the results obtained for Py-Ag and Py-Au complexes. The KS-FEDA and SAPT0 data (see Table 1 and Table 3) show a systematic amplification of the short-range attractive terms for Py-Au structures, especially $E^{pol}$ and, for the V–complex, $E^{disp}$. This effect is most pronounced when comparing Py-Ag and Py-Au in the vertex geometry: the Au complex exhibits a markedly larger (in absolute value) $E^{pol}$, together with a slight absolute increase in $E^{disp}$ (however, not associated with a larger percentage) compared to Ag. This finding can be rationalized by considering that Au is associated with a larger metal polarizability, which directly affects $E^{pol}$. At the same time, both $E^{ele}$ (more attractive) and $E^{ex-rep}$ (more repulsive) increase substantially on Au. This can be ascribed to geometric reasons. In fact, the equilibrium N–metal distances are shorter for Py-Au than for Py-Ag in both morphologies, yielding enhanced Pauli repulsion and near-field electrostatics. This interplay between metal polarizability and equilibrium distance explains why Py-Au exhibits larger energy component magnitudes than Py-Ag yet preserves the same binding physics.

To conclude, we note that, in both systems, the vertex configuration is preferred at all levels, with the exception of SAPT0 in Py-Au complexes for the reasons discussed above. This finding is relevant for optical and photo-catalysis applications [19,121,122,123,124]. In fact, the vertex position is generally associated with the so-called “hot-spots”, where the electric field induced by plasmon excitation can be enhanced by several orders of magnitude and localized in subnanometer volumes [25,26,125,126,127]. Also, vertex hot-spots coincide with the regions that can potentially maximize hot-carrier (electron or hole) injection pathways relevant to plasmonic photo-catalysis [19,128,129,130,131].

## 4. Conclusions

In this work, we have employed the KS-FEDA method to dissect the energetic components governing pyridine adsorption on small silver and gold nanoparticles (Py-Ag20 and Py-Au20). These tetrahedral clusters, viewed as fragments of the face-centered cubic (fcc) metal lattice, provide two prototypical coordination motifs: the S-complex, mimicking adsorption on a (111) surface, and the V-complex, representing a vertex (adatom-like) site. Together, they capture two limiting regimes of molecule–metal bonding—extended surface contact dominated by dispersion and long-range polarization, and localized coordination dominated by short-range electrostatics and induction.

KS-FEDA enables a rigorous and physically transparent decomposition of the interaction energy into its main components, while retaining a full quantum-mechanical description of the metal cluster. The analysis shows that the jorge-DZP basis set reproduces the reference jorge-TZP results within chemical accuracy, thus representing an efficient and reliable choice for larger metallic systems. KS-FEDA results are obtained by exploiting range-separated hybrid functionals with empirical dispersion (CAM-B3LYP-D4) and with NLC functionals (SCAN-rVV10), showing that nearly identical interaction energies and consistent hierarchies of energy components are obtained. For consistency, KS-FEDA results are also compared with SAPT0, which recovers the same physical trends, differing mainly in the distribution between $E^{pol}$ and $E^{disp}$, yet producing similar total interaction energies. The only notable exception is given by Py-Au, for which the short molecule–metal distance and electron correlation, which is only partially captured by SAPT0, result in a SAPT0 overestimation of the interaction energy.

Our results show that, for both metals, adsorption is stronger at the vertex site, where localized electrostatics and induction dominate, while the S-complex benefits more from dispersion interactions. A direct comparison between Py-Ag and Py-Au reveals systematic structure–property patterns. In fact, the amplification of the short-range attractive channels for gold, in particular for $E^{pol}$ and $E^{disp}$, mainly arises from the higher polarizability and greater electron richness of Au, which amplify charge redistribution and near-field interactions. At the same time, the shorter N–Au distances (2.23–2.33 Å) yield larger $E^{ele}$ (more attractive) and $E^{ex-rep}$ (more repulsive).

Overall, KS-FEDA provides a coherent and computationally efficient framework for interpreting molecule–metal bonding in terms of well-defined physical components. It achieves SAPT-like insight directly from DFT calculations, offering a practical route for characterizing adsorption energetics. To extend these analyses to larger and more realistic systems, future developments should combine KS-FEDA with basis sets including effective core potentials (ECPs) and with multilevel DFT [99,132] or embedding schemes [133,134,135], allowing different regions of the system to be treated at variable levels of accuracy. In addition, the physically grounded decomposition offered by KS-FEDA provides a valuable foundation for the parameterization of polarizable or fragment-based force fields [136,137,138] to be exploited for accurate molecule–nanoparticle phase-space sampling [139,140,141,142]. Finally, it is worth remarking that, in this work, KS-FEDA has been applied to non-covalently bonded systems; however, differently from most EDA schemes, the method is general enough to be applied to chemisorbed systems [69,70].

## Figures and Tables

**Table 1:** KS-FEDA energy terms (see Equation (15)) for Py-Ag S- and V-complexes at the CAM-B3LYP-D4 and SCAN-rVV10 levels. SAPT0 and BP86 (obtained using the extended transition state method at the BP86/TZP level of theory from Ref. [87]) results are also reported as a comparison. All energy terms are given in kcal/mol.

	S-Complex				V-Complex			
	CAM-B3LYP-D4	SCAN-rVV10	SAPT0	BP86 [87]	CAM-B3LYP-D4	SCAN-rVV10	SAPT0	BP86 [87]
$E^{ele}$	−22.26	−21.54	−20.33	−23.37	−36.17	−34.82	−33.34	−35.81
$E^{ex-rep}$	32.51	31.63	30.28	30.64	39.62	37.42	35.82	38.15
$E^{pol}$	−8.96	−9.03	−7.14	−9.26	−13.57	−13.75	−11.18	−11.53
$E^{disp}$	−14.10	−13.12	−14.74	–	−7.87	−7.98	−8.89	–
$E^{int}$	−12.81	−12.06	−11.93	−1.99	−17.99	−19.13	−17.59	−9.19

**Table 2:** $E^{ele}$, $E^{pol}$, and $E^{disp}$ relative percentages with respect to the total attractive energy ($E^{ele}+E^{pol}+E^{disp}$) for Py-Ag and Py-Au S- and V-complexes calculated at the KS-FEDA (CAM-B3LYP-D4 and SCAN-rVV10) and SAPT0 levels.

		S-Complex			V-Complex		
		CAM-B3LYP-D4	SCAN-rVV10	SAPT0	CAM-B3LYP-D4	SCAN-rVV10	SAPT0
Py-Ag	$E^{ele}$	49%	49%	48%	63%	62%	62%
	$E^{pol}$	20%	21%	17%	24%	24%	21%
	$E^{disp}$	31%	30%	35%	14%	14%	17%
Py-Au	$E^{ele}$	64%	63%	64%	70%	68%	69%
	$E^{pol}$	21%	23%	16%	22%	23%	17%
	$E^{disp}$	14%	14%	20%	8%	9%	14%

**Table 3:** KS-FEDA energy terms (Equation (15)) for Py-Au S- and V-complexes at the CAM-B3LYP-D4 and SCAN-rVV10 levels. SAPT0 and BP86 (obtained at the BP86/TZP + ZORA level of theory from Ref. [89]) results are also reported for comparison. All energy terms are given in kcal/mol.

	S-Complex				V-Complex			
	CAM-B3LYP-D4	SCAN-rVV10	SAPT0	BP86 [89]	CAM-B3LYP-D4	SCAN-rVV10	SAPT0	BP86 [89]
$E^{ele}$	−64.21	−61.17	−59.61	–	−83.79	−80.14	−73.39	–
$E^{ex-rep}$	87.74	84.45	75.47	–	105.75	100.87	92.19	–
$E^{pol}$	−21.35	−21.82	−14.55	–	−26.76	−27.30	−18.03	–
$E^{disp}$	−14.33	−13.70	−18.61	–	−9.52	−10.22	−14.43	–
$E^{int}$	−12.14	−12.24	−17.30	−5.54	−14.31	−16.78	−13.65	−17.13

## Data Availability

All the data are provided within the paper.

## Supplementary Materials

The following supporting information can be downloaded at: https://www.mdpi.com/article/10.3390/nano15221720/s1. Section S1: KS-FEDA data for Py-Ag; Section S2: KS-FEDA data for Py-Au.
