# Supplementary material for: On the Energy Contributions Driving Pyridine Adsorption on Silver and Gold Nanoparticles
Source: Nanomaterials (Basel). 2025 Nov 13;15(22):1720. doi: 10.3390/nano15221720 (PMC12655558; doi:10.3390/nano15221720)
Supplement: Supplementary file 1 [file nanomaterials-15-01720-s001.zip › nanomaterials-3979329-supplementary.pdf]

Supplementary Materials for: On the Energy Contributions Driving Pyridine Adsorption on Silver and Gold Nanoparticles

Tommaso Giovannini*

Department of Physics, University of Rome Tor Vergata, Via della Ricerca Scientifica 1, I-00133 Rome, Italy

E-mail: tommaso.giovannini@uniroma2.it

S1 Py-Ag

S1.1 S-complex

Table S1: KS-FEDA (Eq. 13) of Py-Ag in S-complex morphology as a function of the DFT functional (CAM-B3LYP-D4 and SCAN-rVV10) and basis set (dGauss-DZVP, jorge-DZP, and jorge-TZP). Absolute energy differences with respect to jorge-TZP values are given in parenthesis. All energies are reported in kcal/mol.

	CAM-B3LYP-D4			SCAN-rVV10		
Energy Term	dGauss-DZVP	jorge-DZP	jorge-TZP	dGauss-DZVP	jorge-DZP	jorge-TZP
E^{int}	-11.61 (0.93)	-12.81 (0.27)	-12.54	-10.73 (0.97)	-12.06 (0.36)	-11.70
E^{ele}	-21.88 (0.36)	-22.26 (0.74)	-21.52	-20.77 (0.35)	-21.54 (1.12)	-20.42
E^{ex}	-46.10 (0.60)	-47.53 (2.03)	-45.50	-45.53 (0.72)	-47.88 (3.07)	-44.81
$E^{corr}_{(0)}$	8.69 (0.20)	8.91 (0.42)	8.49	15.40 (0.44)	16.57 (1.61)	14.96
$\Delta E^{ele}_{\text{ASN}}$	-6.33 (0.27)	-7.29 (0.69)	-6.60	-6.61 (0.33)	-7.65 (0.71)	-6.94
$\Delta E^{HF,x}_{\text{ASN}}$	31.75 (0.53)	32.92 (1.70)	31.22	31.31 (0.58)	33.23 (2.50)	30.73
$E^{el-prep}_{\text{ASN}}$	52.28 (0.32)	54.41 (2.45)	51.96	50.82 (0.24)	53.93 (3.35)	50.58
$\Delta E^{corr}_{\text{ASN}}$	-13.40 (0.13)	-13.87 (0.60)	-13.27	-25.61 (0.39)	-27.13 (1.91)	-25.22
$\Delta E^{ele}_{\text{orb}}$	-16.80 (1.92)	-19.75 (1.03)	-18.72	-23.67 (2.57)	-27.86 (1.62)	-26.24
$\Delta E^{HF,x}_{\text{orb}}$	-7.47 (1.09)	-9.62 (1.06)	-8.56	-11.65 (1.53)	-14.43 (1.25)	-13.18
$\Delta E^{el-prep}_{\text{orb}}$	16.81 (2.45)	20.41 (1.15)	19.26	27.99 (3.61)	33.26 (1.66)	31.60
$\Delta E^{corr}_{\text{orb}}$	-0.52 (0.12)	-0.50 (0.14)	-0.64	-2.42 (0.35)	-2.56 (0.21)	-2.77
E^{disp}		-8.640		–	–	–

Table S2: KS-FEDA (Eq. 15) of Py-Ag in S-complex morphology as a function of the DFT functional (CAM-B3LYP-D4 and SCAN-rVV10) and basis set (dGauss-DZVP, jorge-DZP, and jorge-TZP). All energies are reported in kcal/mol.

	CAM-B3LYP-D4			SCAN-rVV10		
Energy Term	dGauss-DZVP	jorge-DZP	jorge-TZP	dGauss-DZVP	jorge-DZP	jorge-TZP
E^{ele}	-21.88	-22.26	-21.52	-20.77	-21.54	-20.42
$E^{ex}+E^{rep}$	31.60	32.51	31.07	29.99	31.63	29.56
$\Delta E^{HF}_{\text{orb}}$	-7.46	-8.96	-8.02	-7.33	-9.03	-7.82
$E^{corr}+E^{disp}$	-13.87	-14.10	-14.06	-12.63	-13.12	-13.03

S1.2 V-complex

Table S3: KS-FEDA (Eq. 13) of Py-Ag in V-complex morphology as a function of the DFT functional (CAM-B3LYP-D4 and SCAN-rVV10) and basis set (dGauss-DZVP, jorge-DZP, and jorge-TZP). Absolute energy differences with respect to jorge-TZP values are given in parenthesis. All energies are reported in kcal/mol.

	CAM-B3LYP-D4			SCAN-rVV10		
Energy Term	dGauss-DZVP	jorge-DZP	jorge-TZP	dGauss-DZVP	jorge-DZP	jorge-TZP
E^{int}	-14.83 (1.50)	-17.99 (1.66)	-16.33	-16.00 (1.64)	-19.13 (1.49)	-17.64
E^{ele}	-34.33 (0.39)	-36.17 (2.23)	-33.94	-32.51 (0.16)	-34.82 (2.47)	-32.35
E^{ex}	-47.00 (0.36)	-49.48 (2.84)	-46.64	-45.32 (0.07)	-48.62 (3.23)	-45.39
$E^{corr}_{(0)}$	9.89 (0.18)	10.48 (0.77)	9.71	17.69 (0.12)	19.16 (1.59)	17.57
$\Delta E^{ele}_{\text{ASN}}$	2.10 (0.51)	3.84 (1.23)	2.61	1.34 (0.56)	2.80 (0.90)	1.90
$\Delta E^{HF,x}_{\text{ASN}}$	32.84 (0.42)	35.03 (2.61)	32.42	31.36 (0.03)	34.27 (2.94)	31.33
$E^{el-prep}_{\text{ASN}}$	50.08 (1.03)	50.23 (1.18)	49.05	47.72 (0.56)	48.96 (1.80)	47.16
$\Delta E^{corr}_{\text{ASN}}$	-14.13 (0.12)	-14.75 (0.74)	-14.01	-23.94 (0.04)	-25.58 (1.60)	-23.98
$\Delta E^{ele}_{\text{orb}}$	-22.55 (3.65)	-26.88 (0.68)	-26.20	-27.89 (4.50)	-33.35 (0.96)	-32.39
$\Delta E^{HF,x}_{\text{orb}}$	-7.45 (1.52)	-10.05 (1.08)	-8.97	-10.79 (1.96)	-13.69 (0.94)	-12.75
$\Delta E^{el-prep}_{\text{orb}}$	19.46 (4.03)	23.35 (0.14)	23.49	28.03 (5.19)	33.29 (0.07)	33.22
$\Delta E^{corr}_{\text{orb}}$	-0.50 (0.13)	-0.38 (0.25)	-0.63	-1.67 (0.29)	-1.56 (0.40)	-1.96
E^{disp}		-3.229		–	–	–

Table S4: KS-FEDA (Eq. 15) of Py-Ag in V-complex morphology as a function of the DFT functional (CAM-B3LYP-D4 and SCAN-rVV10) and basis set (dGauss-DZVP, jorge-DZP, and jorge-TZP). All energies are reported in kcal/mol.

	CAM-B3LYP-D4			SCAN-rVV10		
Energy Term	dGauss-DZVP	jorge-DZP	jorge-TZP	dGauss-DZVP	jorge-DZP	jorge-TZP
E^{ele}	-34.33	-36.17	-33.94	-32.51	-34.82	-32.35
$E^{ex}+E^{rep}$	38.02	39.62	37.44	35.09	37.42	34.99
$\Delta E^{HF}_{\text{orb}}$	-10.55	-13.57	-11.69	-10.65	-13.75	-11.92
$E^{corr}+E^{disp}$	-7.97	-7.87	-8.15	-7.92	-7.98	-8.36

S1.3 S- and V-complexes

Table S5: KS-FEDA energy terms (Eq. 15) for Py-Ag S- and V-complexes at the CAM-B3LYP-D4 (CAM) and SCAN-rVV10 (SCAN) levels. SAPT0 values are also reported as a comparison. E^{att} indicates the sum of the attractive energy terms ($E^{ele} + E^{pol} + E^{disp}$), and the relative percentages of each component are given in parentheses. All values are given in kcal/mol.

	S-complex			V-complex		
	CAM-B3LYP-D4	SCAN-rVV10	SAPT0	CAM-B3LYP-D4	SCAN-rVV10	SAPT0
E^{ele}	-22.26 (49%)	-21.54 (49%)	-20.33 (48%)	-36.17 (63%)	-34.82 (62%)	-33.34 (62%)
E^{pol}	-8.96 (20%)	-9.03 (21%)	-7.14 (17%)	-13.57 (24%)	-13.75 (24%)	-11.18 (21%)
E^{disp}	-14.1 (31%)	-13.12 (30%)	-14.74 (35%)	-7.87 (14%)	-7.98 (14%)	-8.89 (17%)
E^{att}	-45.32	-43.69	-42.21	-57.61	-56.55	-53.41

S2 Py-Au

Table S6: KS-FEDA (Eq. 13) of Py-Au in S-complex (left) and V-complex (right) morphologies as a function of the DFT functional (CAM-B3LYP-D4/jorge-DZP and SCAN-rVV10/jorge-DZP). All energies are reported in kcal/mol.

	S-complex		V-complex	
Energy Term	CAM-B3LYP-D4	SCAN-rVV10	CAM-B3LYP-D4	SCAN-rVV10
E^{int}	-12.14	-12.24	-14.31	-16.78
E^{ele}	-64.21	-61.17	-83.79	-80.14
E^{ex}	-97.34	-96.40	-107.39	-105.64
$E^{corr}_{(0)}$	24.55	42.79	28.79	49.26
$\Delta E^{ele}_{\text{ASN}}$	6.03	4.39	24.04	21.77
$\Delta E^{HF,x}_{\text{ASN}}$	71.22	70.62	79.61	78.05
$E^{el-prep}_{\text{ASN}}$	107.83	105.83	109.49	106.68
$\Delta E^{corr}_{\text{ASN}}$	-31.62	-54.46	-35.25	-57.47
$\Delta E^{ele}_{\text{orb}}$	-41.59	-55.53	-54.34	-70.54
$\Delta E^{HF,x}_{\text{orb}}$	-20.16	-28.09	-21.85	-30.20
$\Delta E^{el-prep}_{\text{orb}}$	40.40	61.81	49.43	73.45
$\Delta E^{corr}_{\text{orb}}$	0.64	-2.03	0.70	-2.01
E^{disp}	-7.89	–	-3.75	–

Table S7: KS-FEDA energy terms (Eq. 15) for Py-Au S- and V-complexes at the CAM-B3LYP-D4 (CAM) and SCAN-rVV10 (SCAN) levels. SAPT0 values are also reported as a comparison. E^{att} indicates the sum of the attractive energy terms ($E^{ele} + E^{pol} + E^{disp}$), and the relative percentages of each component are given in parentheses. All values are given in kcal/mol.

	S-complex			V-complex		
	CAM-B3LYP-D4	SCAN-rVV10	SAPT0	CAM-B3LYP-D4	SCAN-rVV10	SAPT0
E^{ele}	-64.21 (64%)	-61.17 (63%)	-59.61 (64%)	-83.79 (70%)	-80.14 (68%)	-73.39 (69%)
E^{pol}	-21.35 (21%)	-21.82 (23%)	-14.55 (16%)	-26.76 (22%)	-27.3 (23%)	-18.03 (17%)
E^{disp}	-14.33 (14%)	-13.7 (14%)	-18.61 (20%)	-9.52 (8%)	-10.22 (9%)	-14.43 (14%)
E^{att}	-99.89	-96.69	-92.77	-120.07	-117.66	-105.85
